# Subjective sleepiness and objective sleep propensity in adults with attention-deficit/hyperactivity disorder referred for multiple sleep latency testing

**DOI:** 10.3389/fpsyt.2026.1848254

**Published:** 2026-06-29

**Authors:** Sunao Uchida, Takashi Maruo, Shunsuke Takagi, Genichi Sugihara, Hidehiko Takahashi

**Affiliations:** 1 Sleep Psychiatry Institute and Sunao Clinic, Docilis Mc, Saitama, Japan; 2 Waseda University, Tokyo, Japan; 3 Department of Psychiatry and Behavioral Sciences, Institute of Science Tokyo, Tokyo, Japan; 4 Center for Brain Integration Research, Institute of Science Tokyo, Tokyo, Japan

**Keywords:** adult ADHD, attention-deficit/hyperactivity disorder, Epworth Sleepiness Scale, excessive daytime sleepiness, hypersomnolence, multiple sleep latency test, sleepiness

## Abstract

**Introduction:**

Adults with attention-deficit/hyperactivity disorder (ADHD) often report excessive daytime sleepiness, but the relationship between subjective sleepiness and objective sleep propensity remains unclear. We examined this relationship in adults referred for Multiple Sleep Latency Test (MSLT) evaluation, using a clinical comparison group with excessive daytime sleepiness (EDS) but without ADHD.

**Methods:**

In this retrospective cross-sectional study, we analyzed medical records of 130 adults aged 18 years or older who underwent MSLT between January and December 2021, including 68 adults in the ADHD group and 62 in the EDS-only group. Subjective sleepiness was assessed by the Epworth Sleepiness Scale (ESS) and objective sleep propensity by mean MSLT sleep latency, with MSLT positivity defined as mean sleep latency ≤ 480 s. Associations between ESS scores and mean sleep latency were assessed within each group, and correlation coefficients were compared between groups using Fisher's r-to-z transformation.

**Results:**

ESS scores did not differ significantly between groups, with median scores of 14.0 in the ADHD group and 13.0 in the EDS-only group. In contrast, objective sleep propensity differed significantly: median mean sleep latency was longer in the ADHD group than in the EDS-only group (432.0 s vs 322.0 s, p = 0.008), and MSLT positivity was less frequent in the ADHD group (61.8% vs 87.1%, p = 0.001). Within the ADHD group, ESS scores were not significantly correlated with mean sleep latency, including among MSLT-positive cases. A significant inverse correlation was observed in the MSLT-positive EDS-only subgroup, although formal comparison of correlation coefficients did not demonstrate a statistically significant between-group difference in the ESS–MSLT relationship. SOREMP frequencies were numerically higher in the EDS-only group but did not differ significantly between groups.

**Discussion:**

These findings suggest that subjective sleepiness complaints and objective sleep propensity may not closely align in adults with ADHD referred for sleep evaluation, and support the need for integrated psychiatric and sleep-medicine assessment when such patients present with excessive daytime sleepiness.

## Introduction

Attention-deficit/hyperactivity disorder (ADHD) is a neurodevelopmental disorder characterized by persistent symptoms of inattention, hyperactivity, and impulsivity that can continue into adulthood (1–3). Sleep-related complaints are common in adults with ADHD, and excessive daytime sleepiness (EDS) is clinically important because it may impair daytime functioning and overlap with fatigue, attentional difficulty, and symptoms of central hypersomnolence (4).

Daytime sleepiness is commonly assessed using both subjective and objective measures. The Epworth Sleepiness Scale (ESS) is widely used to assess self-reported sleepiness in everyday situations (5), whereas the Multiple Sleep Latency Test (MSLT) is the standard physiological measure of daytime sleep propensity and is central to the evaluation of disorders such as narcolepsy and idiopathic hypersomnia (6).

In adults with ADHD, subjective sleep complaints are consistently reported, but objective findings have been less consistent. A meta-analysis by Díaz-Román et al. (7) found that subjective sleep problems were associated with ADHD in adults, whereas objective sleep measures showed less consistent abnormalities. This pattern raises the possibility that subjective sleepiness and objective sleep propensity may not closely align in ADHD.

Direct evidence remains limited. In a pilot MSLT study, Sobanski et al. (8) found higher subjective sleepiness in adults with ADHD without a corresponding difference in mean sleep latency. Using the Maintenance of Wakefulness Test, Bioulac et al. (9) similarly reported that a substantial proportion of adults with ADHD showed preserved objective alertness despite subjective complaints of sleepiness. Bioulac et al. (10) later reported that objectively sleepy adults with ADHD did not show clearly altered homeostatic sleep-pressure buildup under controlled conditions. Experimental work has also shown that sleep-like slow waves can intrude into wakefulness and predict attentional lapses (11), suggesting that subjective sleepiness may arise through mechanisms not fully captured by standard objective measures of physiological sleep propensity.

Despite the clinical relevance of this issue, systematic investigation of the relationship between subjective and objective sleepiness in adults with ADHD remains limited, especially in real-world clinical populations referred for sleep evaluation. The present study examined this issue in a retrospective clinical cohort of adults who underwent MSLT. Our primary objective was to assess the relationship between subjective sleepiness, measured by the ESS, and objective sleep propensity, measured by mean MSLT sleep latency, in adults in the ADHD group and to compare this relationship with that observed in a clinical comparison group with EDS but without ADHD. We hypothesized that adults in the ADHD group would show a weaker and less consistent association between ESS scores and MSLT findings than the EDS-only group.

## Methods

### Study design and setting

This retrospective cross-sectional study was conducted at Sunao Clinic, Saitama, Japan. The study was approved by the Ethics Committee of Tokyo Medical and Dental University (now Institute of Science Tokyo). Because of the retrospective design and use of existing medical records, the requirement for informed consent was waived.

### Participants

We reviewed the records contained in the analytic screening dataset for patients who underwent MSLT in 2021. For the present analysis, eligible cases were restricted to adults aged 18 years or older whose category was coded as ADD, ADHD, or EDS only in the source clinical dataset. In the source clinical dataset, the labels “ADD” and “ADHD” were historical clinical coding labels used in routine practice. Specifically, “ADD” was used for cases corresponding to predominantly inattentive presentation, whereas “ADHD” was used for cases corresponding to combined presentation; no cases were coded separately for hyperactive-impulsive presentation alone. Because the present study was not designed to compare ADHD presentations, cases coded as ADD and ADHD in the source dataset were combined analytically and are referred to throughout the manuscript as the ADHD group. The comparison group consisted of patients coded as EDS only, a designation that reflects the absence of ADHD rather than a specific or homogeneous diagnosis.

Exclusion criteria included individuals younger than 18 years and records with non-target diagnostic categories, including other primary psychiatric disorders such as mood or anxiety disorders, autism spectrum disorder, and obstructive sleep apnea. The screening dataset contained 195 records. After exclusion of 38 records outside the prespecified diagnostic categories (Exclude, n = 29; ASD, n = 9) and 27 individuals younger than 18 years, 130 adults remained for analysis, including 68 in the ADHD group and 62 in the EDS-only group.

ADHD was diagnosed by psychiatrists experienced in adult ADHD on the basis of comprehensive clinical evaluation using DSM-5 criteria (1). Diagnosis was based on clinical interview and review of available developmental and clinical information obtained from patients and, when available, from parents or other collateral sources and records.

### Measures

The Epworth Sleepiness Scale (ESS) is an 8-item self-report questionnaire that assesses the likelihood of dozing in common daily situations (5). Total scores range from 0 to 24, with higher scores indicating greater subjective daytime sleepiness.

The Multiple Sleep Latency Test (MSLT) was performed according to standard American Academy of Sleep Medicine recommendations after nocturnal polysomnography (6). Four or five nap opportunities were scheduled at 2-hour intervals, and mean sleep latency was calculated as the average across nap opportunities. For subgroup analyses, MSLT positivity was defined as mean sleep latency ≤ 480 s (≤ 8 minutes). SOREMP counts per participant were also recorded from the clinical dataset, defined as the number of nap opportunities in which REM sleep onset occurred within 15 minutes.

Patients taking medications that could affect sleep or wakefulness were instructed, when clinically feasible, to discontinue or reduce those medications for two weeks before testing. No participants were taking ADHD medication at the time of MSLT.

### Statistical analysis

Continuous variables were summarized as median and interquartile range (IQR) and compared between groups using two-sided Mann–Whitney U tests. Categorical variables were summarized as counts and percentages. Group differences in MSLT positivity and SOREMP frequency were examined using two-sided Fisher’s exact test. Associations between subjective sleepiness and objective sleep propensity were assessed separately within each group using Pearson correlations between ESS scores and mean MSLT sleep latency in seconds; Spearman correlations were examined as a sensitivity analysis. Analyses involving ESS used complete-case analysis, with nonnumeric ESS values treated as missing. Differences between correlation coefficients across groups were assessed using Fisher’s r-to-z transformation. All tests were two-sided, and p values less than 0.05 were considered statistically significant. This study was retrospective and exploratory in nature and was not preregistered.

## Results

### Participant characteristics

The final analytic sample comprised 130 adults, including 68 in the ADHD group and 62 in the EDS-only group. Sex distribution was similar between groups, and the median age was in the mid-twenties in both groups (**Table 1**).

**Table 1:** Demographic characteristics by group.

Group	n	Female, n (%)	Male, n (%)	Age, median [IQR], years
ADHD	68	46 (67.6)	22 (32.4)	26.71 [23.42–31.62]
EDS-only	62	33 (53.2)	29 (46.8)	27.42 [23.35–32.13]

### Subjective sleepiness and objective sleep propensity

ESS scores did not differ significantly between groups (**Table 2**). Median ESS was 14.0 [11.0–17.0] in the ADHD group and 13.0 [11.0–15.0] in the EDS-only group, with two missing ESS values in the ADHD group. These findings indicate that the two groups reported comparably elevated levels of subjective daytime sleepiness.

**Table 2:** Subjective and objective sleepiness by group.

Measure	ADHD, median [IQR] (N)	EDS-only, median [IQR] (N)	p value
ESS	14.0 [11.0–17.0] (66)	13.0 [11.0–15.0] (62)	0.395
Mean MSLT sleep latency, s	432.0 [268.5–638.0] (68)	322.0 [178.0–435.5] (62)	0.008

Objective sleep propensity differed between groups. Median mean MSLT sleep latency was longer in the ADHD group than in the EDS-only group (432.0 s [268.5–638.0] vs 322.0 s [178.0–435.5], p = 0.008), indicating lower physiological sleep propensity in the ADHD group despite similar ESS scores.

### MSLT positivity

Consistent with the longer mean sleep latency in the ADHD group, MSLT positivity was less frequent in the ADHD group than in the EDS-only group (**Table 3a**). Using the prespecified threshold of mean sleep latency ≤ 480 s (≤ 8 min), 42 of 68 participants in the ADHD group (61.8%) were MSLT-positive, compared with 54 of 62 participants in the EDS-only group (87.1%; p = 0.001, Fisher’s exact test; RR = 0.71, 95% CI 0.57–0.87; OR = 0.24, 95% CI 0.10–0.58; RD = −0.253, 95% CI −0.396 to −0.111).

**Table 3A:** MSLT positivity by group.

Group	MSLT-positive, n (%)	MSLT-negative, n (%)
ADHD (n = 68)	42 (61.8)	26 (38.2)
EDS-only (n = 62)	54 (87.1)	8 (12.9)

MSLT positivity was defined as mean sleep latency ≤ 480 s (≤ 8 min). Effect sizes: RR = 0.71 (95% CI 0.57–0.87); OR = 0.24 (95% CI 0.10–0.58); RD = −0.253 (95% CI −0.396 to −0.111).

p = 0.001 (Fisher's exact test, two-sided).

**Table 3B:** Subjective and objective sleepiness in the MSLT-positive subgroup.

Measure	ADHD, median [IQR] (N)	EDS-only, median [IQR] (N)	p value
ESS	15.0 [10.0–17.0] (41)	13.0 [11.0–15.75] (54)	0.493
Mean MSLT sleep latency, s	283.0 [179.5–378.5] (42)	273.0 [167.5–387.0] (54)	0.717

Within the MSLT-positive subgroup, ESS scores and mean sleep latencies did not differsignificantly between groups (**Table 3b**). Median ESS was 15.0 [10.0–17.0] in the ADHD group and 13.0 [11.0–15.75] in the EDS-only group (p = 0.493). Median mean sleep latency was 283.0 s [179.5–378.5] in the ADHD group and 273.0 s [167.5–387.0] in the EDS-only group (p = 0.717).

### Associations between ESS and mean MSLT sleep latency

In the ADHD group, ESS was not significantly associated with mean MSLT sleep latency in the full sample (Pearson r = −0.049, p = 0.699, n = 66). When the analysis was restricted to MSLT-positive participants, the ESS–MSLT association in the ADHD group remained non-significant (r = −0.246, p = 0.122, n = 41).

In the EDS-only group, ESS showed a non-significant inverse association with mean MSLT sleep latency in the full sample (r = −0.205, p = 0.110, n = 62). In the MSLT-positive EDS-only subgroup, this inverse association reached statistical significance (r = −0.278, p = 0.042, n = 54).

Formal comparison of correlation coefficients using Fisher’s r-to-z transformation showed no significant between-group difference in the ESS–MSLT association in either the full sample (z = 0.880, p = 0.379) or the MSLT-positive subgroup (z = 0.163, p = 0.870). Thus, although the inverse association reached statistical significance only in the MSLT-positive EDS-only subgroup, the strength of the correlations did not differ significantly between groups.

**Figure 1:**
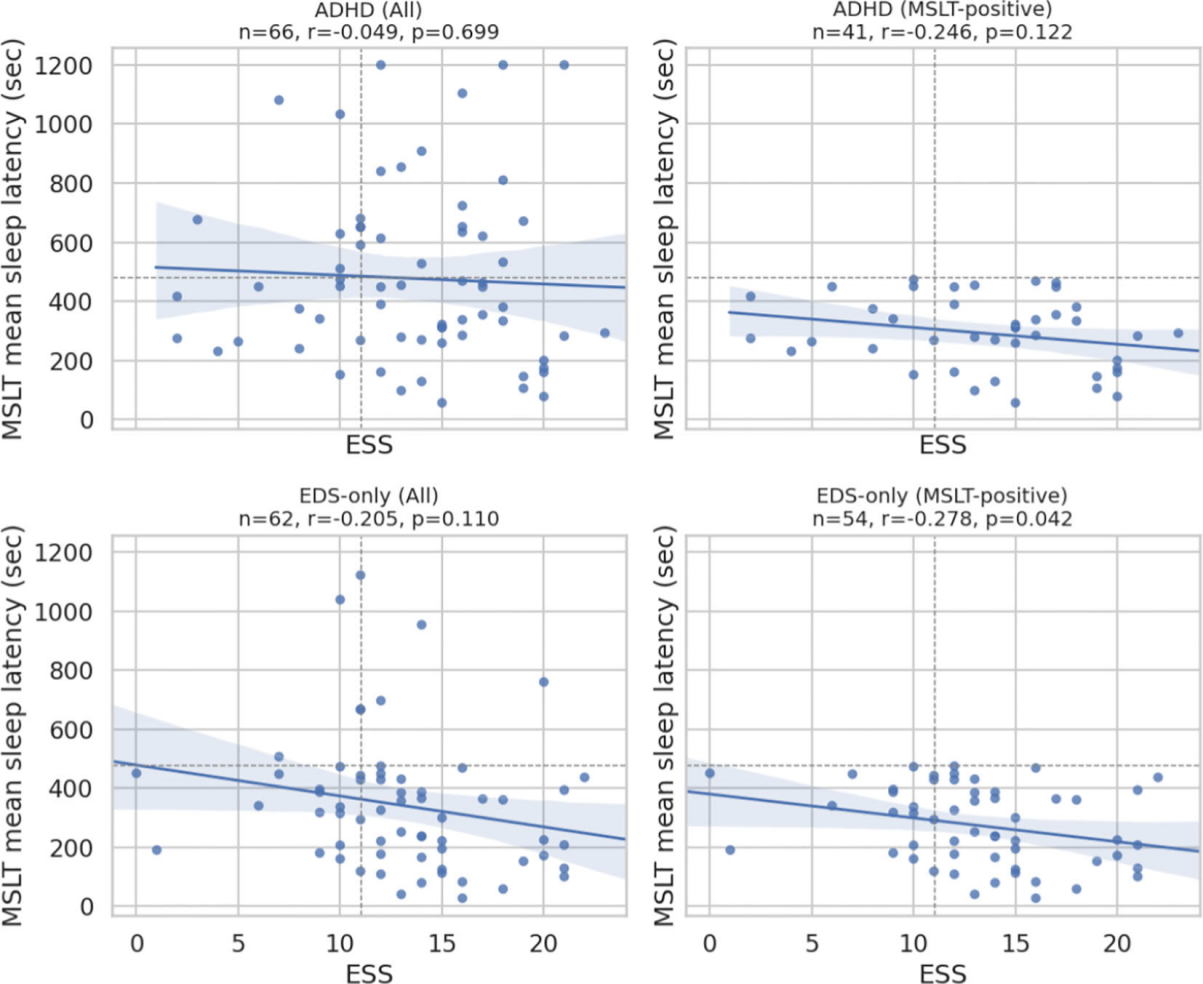
Associations between ESS score and mean MSLT sleep latency in the ADHD and EDS-only groups, shown for the full cohort (left panels) and the MSLT-positive subgroup (right panels). Dashed horizontal line indicates the MSLT positivity threshold (480 s). Shaded areas represent 95% confidence intervals of the regression line.

**Figure 2:**
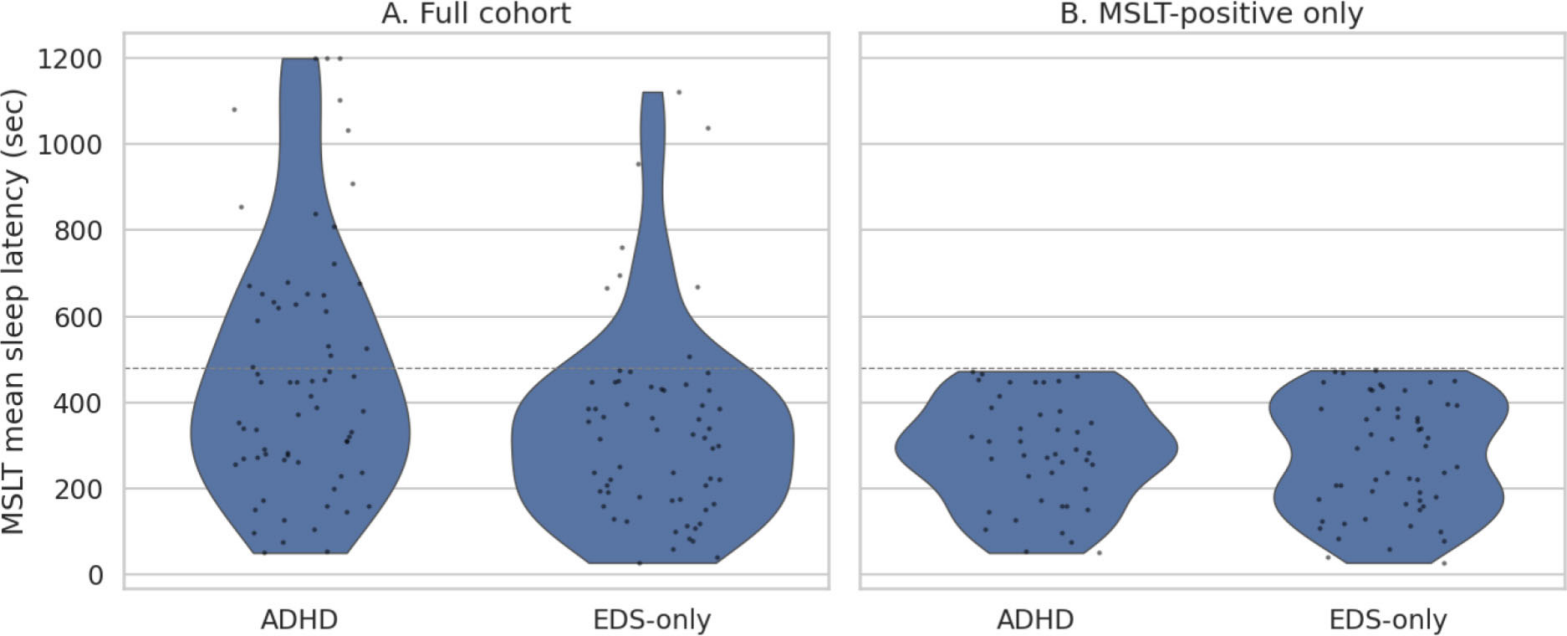
Distributions of mean MSLT sleep latency in the ADHD and EDS-only groups in the full cohort **(A)** and in the MSLT-positive subgroup **(B)**. Dashed horizontal line indicates the MSLT positivity threshold (480 s). Individual data points are shown.

As shown in **Figure 1**, the scatterplots suggest a weak ESS–latency relationship in the ADHD group in both the full cohort and the MSLT-positive subset, whereas the inverse trend is more apparent in the MSLT-positive EDS-only subgroup. **Figure 2** likewise shows that mean MSLT sleep latency was longer in the ADHD group than in the EDS-only group in the full cohort, whereas median latencies were similar between groups within the MSLT-positive subgroup.

### SOREMP findings

SOREMP counts were reviewed for all participants in the analytic sample (**Table 4**). In the ADHD group (n = 68), the distribution of SOREMP counts was: 0 SOREMPs in 50 participants (73.5%), 1 SOREMP in 6 (8.8%), 2 SOREMPs in 10 (14.7%), 3 SOREMPs in 1 (1.5%), and 4 SOREMPs in 1 (1.5%), giving a mean of 0.49. In the EDS-only group (n = 62), the distribution was: 0 SOREMPs in 39 participants (62.9%), 1 SOREMP in 6 (9.7%), 2 SOREMPs in 10 (16.1%), 3 SOREMPs in 4 (6.5%), and 4 SOREMPs in 3 (4.8%), giving a mean of 0.81. The proportion of participants with at least one SOREMP was 18/68 (26.5%) in the ADHD group and 23/62 (37.1%) in the EDS-only group (p = 0.257, Fisher’s exact test). The proportion with at least two SOREMPs was 12/68 (17.6%) in the ADHD group and 17/62 (27.4%) in the EDS-only group (p = 0.210). Within the MSLT-positive subgroup, the proportion with at least two SOREMPs was 11/42 (26.2%) in the ADHD group and 17/54 (31.5%) in the EDS-only group. These between-group differences in SOREMP frequency were not statistically significant. Because individual REM sleep latency values across nap opportunities were not systematically available for retrospective extraction, REM latency analyses were not performed.

**Table 4:** SOREMP distribution by group.

SOREMP count	ADHD, n (%)	EDS-only, n (%)
0	50 (73.5)	39 (62.9)
1	6 (8.8)	6 (9.7)
2	10 (14.7)	10 (16.1)
3	1 (1.5)	4 (6.5)
4	1 (1.5)	3 (4.8)
Mean	0.49	0.81
≥1 SOREMP, n (%)	18 (26.5)	23 (37.1)
≥2 SOREMPs, n (%)	12 (17.6)	17 (27.4)

p values (Fisher’s exact test): ≥1 SOREMP p, 0.257; ≥2 SOREMPs p, 0.210.

## Discussion

This study examined the relationship between subjective and objective measures of sleepiness in adults with ADHD using a retrospective clinical cohort referred for MSLT evaluation. Participants in the ADHD and EDS-only groups reported similar levels of subjective sleepiness on the ESS, whereas the ADHD group showed significantly longer mean MSLT sleep latencies and a lower frequency of MSLT positivity. Within the ADHD group, ESS scores were not significantly associated with mean MSLT sleep latency. In contrast, in the MSLT-positive EDS-only subgroup, a modest inverse association was observed. However, formal comparison of correlation coefficients did not demonstrate a statistically significant between-group difference in the ESS–MSLT relationship. Accordingly, the present findings should not be interpreted as establishing a distinct correlation structure in ADHD relative to the comparison group. Rather, they indicate that in this clinical ADHD group, subjective sleepiness complaints did not consistently align with objective MSLT sleep propensity.

### Interpretation of findings

One possible interpretation is that subjective sleepiness complaints in adults with ADHD partly reflect attentional difficulty, mental fatigue, or broader arousal dysregulation rather than physiological sleep propensity alone. This interpretation is broadly consistent with prior literature showing stronger subjective than objective sleep abnormalities in adults with ADHD (7–10). Related clinical observations have also been reported; Ito et al. (12) described a hypersomnia subgroup with ADHD features and discussed its possible relationship to narcolepsy type 2.

Altered sleep regulation may also contribute to the observed pattern, although the present dataset does not allow direct mechanistic inference. Bioulac et al. (10) examined a small drug-free subgroup of sleepy adults with ADHD under controlled conditions and reported reduced ability to maintain wakefulness without clear evidence that this was explained by altered homeostatic sleep-pressure buildup. This prior work supports the view that daytime sleepiness complaints in ADHD may arise through mechanisms not fully captured by standard physiological sleep propensity measures alone.

Circadian and sleep-schedule factors may also influence the interpretation of objective hypersomnolence testing. Recent evidence suggests that delayed sleep-wake schedules can affect MSLT-based differentiation among central hypersomnolence disorders (13), highlighting the importance of considering sleep timing when interpreting MSLT findings in adults with ADHD.

### Clinical implications

These findings have several practical implications for clinicians evaluating sleep complaints in adults with ADHD. Subjective reports of “sleepiness” should be interpreted cautiously, because they may reflect true hypersomnolence in some patients but in others may reflect attentional difficulty, fatigue, arousal instability, or mixed phenomena. Clinicians should therefore distinguish excessive sleepiness from inattention, low energy, burnout, and other causes of impaired daytime functioning, with particular attention to the developmental timing of symptoms: inattentiveness beginning in childhood without pathological sleepiness points toward primary ADHD, whereas sleepiness and inattentiveness emerging together in adolescence or adulthood may indicate a sleep-wake disorder.

Objective testing with MSLT remains essential for diagnosing central disorders of hypersomnolence. In our cohort, 38.2% of participants in the ADHD group were MSLT-negative despite elevated ESS scores, indicating that a substantial minority would not meet objective criteria for pathological sleep propensity. Sleepiness complaints in adults with ADHD should therefore not automatically be assumed to reflect primary hypersomnolence, and differential diagnoses—including the possibility that fatigue or hypersomnia arises partly in the context of psychological stress or trauma—should be considered (14). When comorbid sleep disorders are present alongside ADHD, they require specific evaluation and treatment in their own right, and psychiatric and sleep-focused interventions are often complementary rather than mutually exclusive. This point is particularly relevant because stimulant medications such as methylphenidate may influence both attentional functioning and daytime alertness, so longitudinal follow-up is important when interpreting treatment response (15).

### Strengths and limitations

This study has several strengths. It included a relatively large sample for MSLT-based research in adults with ADHD, used objective sleep testing in a real-world clinical setting, and directly examined the relationship between subjective and objective measures rather than comparing group means alone. The use of an EDS-only clinical comparison group provides a more stringent and clinically relevant contrast than comparison with healthy volunteers.

Several limitations should be acknowledged. First, the retrospective design precludes causal inference and may introduce referral-related selection bias, as all participants had already been referred for MSLT. Second, although patients were instructed to maintain adequate sleep before testing, objective verification of sleep duration and quality in the preceding weeks was limited; factors such as sleep schedule regularity and circadian timing were not systematically assessed, and these are particularly relevant in ADHD populations where delayed or irregular sleep-wake patterns are common. Third, the EDS-only comparison group was clinically heterogeneous and likely included patients with a range of central disorders of hypersomnolence, such as narcolepsy type 2 and idiopathic hypersomnia, as well as other sleep-wake disorders; the diagnostic composition of this group was not further characterized in the present dataset, which limits the specificity of between-group comparisons. The high MSLT positivity rate (87.1%) in the EDS-only group is consistent with the presence of central hypersomnolence disorders in a substantial proportion of these patients. Fourth, although patients were instructed to discontinue or reduce medications that could affect sleep or wakefulness for two weeks before testing and no participants were taking ADHD medication at the time of MSLT, complete medication washout was not verified and participants were not required to be medication-naive; residual effects of other psychotropic or sleep-related medications, including antidepressants, antipsychotics, sedative-hypnotics, and antihistamines, on MSLT outcomes and subjective sleepiness ratings cannot be excluded. Finally, although the MSLT-positive EDS-only subgroup showed a statistically significant inverse ESS–MSLT correlation, the Fisher r-to-z test did not show a significant difference in correlation strength between groups; the present findings therefore support inconsistency between subjective complaints and objective sleep propensity within the ADHD group, but should not be overinterpreted as demonstrating a definitively different correlation structure between ADHD and EDS-only patients.

### Future directions

Future studies should prospectively examine subjective sleepiness, objective sleep propensity, sleep schedule, and circadian timing in adults with ADHD referred for sleep evaluation. Such work may help clarify which patients show closer alignment between subjective and objective measures and which do not. It will also be important to determine whether treatment of ADHD symptoms, circadian misalignment, or coexisting sleep disorders changes the relationship between ESS scores and MSLT findings over time.

## Conclusions

This study examined subjective sleepiness and objective sleep propensity in adults with ADHD referred for MSLT. Compared with the EDS-only group, adults with ADHD reported similar levels of subjective sleepiness on the ESS but showed longer mean MSLT sleep latencies and a lower frequency of MSLT positivity. Within the ADHD group, ESS scores were not significantly associated with mean MSLT sleep latency, and SOREMP frequencies did not differ significantly between groups. These findings suggest that sleepiness complaints in adults with ADHD may reflect attentional difficulty, arousal dysregulation, or other ADHD-related phenomena rather than physiological sleep propensity alone. Clinicians should interpret sleep complaints in adults with ADHD cautiously and consider comprehensive evaluation, including objective testing when appropriate.

## Data Availability

The data analyzed in this study is subject to the following licenses/restrictions: This study was retrospective and exploratory in nature and was not preregistered. Requests to access these datasets should be directed to the corresponding author, Sunao Uchida (sunao@waseda.jp). The datasets generated and analyzed for this study consist of retrospective clinical records from adult patients who underwent Multiple Sleep Latency Testing at Sunao Clinic in 2021. These data contain potentially identifiable health information and are therefore not publicly available due to institutional and ethical restrictions. De−identified data underlying the main findings (including the analytic dataset used for the reported statistics) will be made available by the corresponding author to qualified researchers upon reasonable request, conditional on approval by the relevant institutional ethics committee and the data−holding clinic.
